# Role of exotic felids as carriers of multidrug-resistant *Salmonella* Newport

**DOI:** 10.1128/asmcr.00227-25

**Published:** 2026-07-01

**Authors:** Francisco A. Uzal

**Affiliations:** 1 California Animal Health and Food Safety Laboratory, San Bernardino Branch, School of Veterinary Medicine, University of California-Davis 115240, Davis, California, USA; Pattern Bioscience, Austin, Texas, USA

**Keywords:** multidrug resistance, Pallas’s cat, persisting strain REPJJP01, salmonellosis, *Salmonella enterica *serovar Newport, snow leopard

## Abstract

Chronic salmonellosis in exotic felids is uncommon. In a recent article in *ASM Case Reports* (2:e00098-25, 2025, https://doi.org/10.1128/asmcr.00098-25), B. L. S. Stenger, D. R. Evans, S. J. Gefroh, A. Breuer, et al. published a report on multidrug-resistant (MDR) *Salmonella enterica* serovar Newport, persisting strain REPJJP01 isolated from two snow leopards (*Panthera uncia*) and a Pallas’s cat (*Otocolobus manul*) in two separate regional zoos. *Salmonella* Newport causes human and animal enteric disease, but this particular strain has not been isolated from felids before. The reported cases highlight the value of genotypic and phenotypic techniques to identify shared and potentially MDR strains of *Salmonella* from human and animal hosts.

## COMMENTARY

Salmonellosis occurs in humans and animals, and transmission between species occurs frequently. With multidrug-resistant (MDR) strains of *Salmonella* spp. becoming more common, it is important to have surveillance information about *Salmonella* serotype carriage in animals, as these can be a source of infection for humans and other animals. While there is abundant information about *Salmonella* serotypes in production animals (e.g., cattle, pigs, horses, etc.), information about the role of this microorganism in disease or death of felids is scant (1–6). A suspected source of infection for exotic felids is raw meat, which poses a risk to other animals fed the same diet and caretakers (1–4). Among felids, less information is available about salmonellosis in Pallas’s cats (*Otocolobus manul*) and snow leopards (*Panthera uncia*). The Pallas’s cat is a small wild cat native to most of Asia. The snow leopard (*P. uncia*) is a large cat native to the mountain ranges of Central and South Asia. Both species are also found in several zoological collections around the world. Although the paucity of previous reports of salmonellosis in these species makes it tempting to speculate that the condition is uncommon in Pallas’s cats and snow leopards, it is difficult to say conclusively whether this is the case, as diagnostic reports and surveillance results have not been published. In this regard, it must be kept in mind that, contrary to what occurs in human medicine, where there is an abundance of data on most infectious diseases affecting the human population around the world, veterinary medicine has several serious limitations in gathering information on these diseases, particularly in zoo and wild animal populations, the health of which is not routinely evaluated.

Stenger and colleagues reported for the first time MDR *Salmonella enterica* serovar Newport persisting strain REPJJP01 in two captive snow leopards and a Pallas’s cat (7). One of the snow leopards had a long history of diarrhea and inappetence followed by death. A post-mortem examination revealed ulcerative colitis, intestinal stricture, and lymphadenomegaly. *S*. Newport was isolated from the intestine of this animal, and the same microorganism was isolated from feces of an asymptomatic enclosure mate. Whole genome sequencing (WGS) of the surviving cage mate isolates revealed multiple antimicrobial resistance (AMR) genes, prompting a retrospective review of previous exotic felid cases received at the laboratory. That effort identified another *S*. Newport strain from a male Pallas’s cat housed in a second regional zoo the prior year. Further characterization of all three isolates (two from snow leopards and one from the Pallas’s cat), including long-read sequencing and hybrid genome assembly of felid genomes, resolved a circularized 47 kb plasmid that was shared by all three genomes. This plasmid carried AMR genes that encoded resistance to aminoglycosides, beta-lactams, trimethoprim, fluoroquinolones, macrolides, phenicols, quaternary ammonium compounds, sulfonamides, and tetracyclines. To match genotype with phenotype, antimicrobial susceptibility testing (AST) was also performed, and minimal inhibitory concentration (MIC) results were identical for all three isolates. These are significant findings as some of these drugs are among the most frequently used antimicrobials in human and veterinary medicine.

Phylogenetic and single nucleotide polymorphism (SNP) analyses of felid isolate genomes and contextual genomes identified from the same subclade resolved by the NCBI SNP Tree Viewer tool (*n* = 158 genomes) confirmed the close genetic relatedness of felid isolate genomes to those from human clinical, meat, animal fecal, and environmental isolates, highlighting the risk of transmission between animals, humans, and the environment.

The reported cases highlight the value of genotypic and phenotypic techniques for the diagnosis and characterization of salmonellosis cases. While phenotyping provides information about microbial susceptibility and other aspects of the isolates, genotypic techniques allowed for a deeper understanding of them and provide valuable information to understand the epidemiology of these infections.

What makes this study novel and interesting is that, in addition to the full characterization of the isolates, a full necropsy and histopathology were performed in one of the snow leopards, and the authors provide the results of an interesting combination of molecular biology and anatomic pathology, something on which there is little information in felids. Among other lesions, Stenger et al. describe an intestinal stricture in the deceased snow leopard; this is an interesting finding that, to the best of our knowledge, has only been described before in pigs with spontaneous salmonellosis (8) and in mice with experimental salmonellosis (9). The other main lesions described during the post-mortem examination of the snow leopard (e.g., ulcerative and fibrinonecrotizing colitis and lymphadenomegaly) are common responses to salmonellosis in humans and other animals (10–13).

Another interesting aspect of this paper is the treatment of the asymptomatic enclosure mate of the dead snow leopard. As the authors explain, treatment of asymptomatic *Salmonella* infections is not recommended due to the potential for prolonged shedding and risk of perpetuating AMR development. However, in this case, treatment was elected due to the value of the endangered animal species involved, a snow leopard. Because treatment of asymptomatic *Salmonella* carriers remains a controversial issue (14), it would have been helpful to get information on the success of the treatment of this animal, something unfortunately not addressed in this manuscript.

This report helps us to understand the risk of animal to human transmission of *Salmonella* spp. and the risk to animal caretakers. The final recommendation provided in this paper—that zoo animal facility workers be educated on appropriate biosecurity for moving between enclosures, proper handling of food, and handwashing procedures—is a critical one, as salmonellosis is a well-known zoonosis and possibly an anthropozoonosis (15). Continued characterization of *Salmonella* strains isolated from animals to help close epidemiological gaps is also recommended. Fortunately, this is currently routinely performed in many veterinary diagnostic laboratories in the United States and elsewhere (14). Surveillance of salmonellosis in wild felids is, however, not something regularly performed, and the results of this paper pose the question of how prevalent salmonellosis is among wild and captive felids, which is an open invitation for researchers to follow.
